# Temporospatial characterization of ventricular wall motion with real-time cardiac magnetic resonance imaging in health and disease

**DOI:** 10.1038/s41598-022-08094-3

**Published:** 2022-03-08

**Authors:** Yu Y. Li, Shams Rashid, Jason Craft, Yang J. Cheng, William Schapiro, Kathleen Gliganic, Ann-Marie Yamashita, Marie Grgas, Elizabeth Haag, J. Jane Cao

**Affiliations:** grid.416387.f 0000 0004 0439 8263 St. Francis Hospital, DeMatteis Center for Research and Education, Cardiac Imaging, 101 Northern Blvd, Greenvale, NY 11548 USA

**Keywords:** Cardiology, Magnetic resonance imaging

## Abstract

Cardiac magnetic resonance imaging (MRI) has been largely dependent on retrospective cine for data acquisition. Real-time imaging, although inferior in image quality to retrospective cine, is more informative about motion dynamics. We herein developed a real-time cardiac MRI approach to temporospatial characterization of left ventricle (LV) and right ventricle (RV) wall motion. This approach provided two temporospatial indices, temporal periodicity and spatial coherence, for quantitative assessment of ventricular function. In a cardiac MRI study, we prospectively investigated temporospatial characterization in reference to standard volumetric measurements with retrospective cine. The temporospatial indices were found to be effective for evaluating the difference of ventricular performance between the healthy volunteers and the heart failure (HF) patients (LV temporal periodicity 0.24 ± 0.037 vs. 0.14 ± 0.021; RV temporal periodicity 0.18 ± 0.030 vs. 0.10 ± 0.014; LV spatial coherence 0.52 ± 0.039 vs. 0.38 ± 0.040; RV spatial coherence 0.50 ± 0.036 vs. 0.35 ± 0.035; all in arbitrary unit). The HF patients and healthy volunteers were well differentiated in the scatter plots of spatial coherence and temporal periodicity while they were mixed in those of end-systolic volume (ESV) and ejection fraction (EF) from volumetric measurements. This study demonstrated the potential of real-time cardiac MRI for intricate analysis of ventricular function beyond retrospective cine.

## Introduction

Magnetic resonance imaging (MRI) is an essential tool for clinical diagnosis of cardiovascular diseases^1,2^. Due to imaging speed limitation, however, cardiac MRI has been largely dependent on retrospective cine for data acquisition. Retrospective cine can collect data from several cardiac cycles with breath-holding and sort them into a virtual cardiac cycle in reference to electrocardiogram (ECG) gating during post processing^3,4^. This technique provides a set of single-cycle multi-phase images for quantitative analysis with an assumption that the heartbeat variation be minor across different cardiac cycles. Owing to its reliable performance, retrospective cine has been used as a primary tool for cardiac MRI data acquisition. Volumetric measurements with retrospective cine have become a clinical standard for quantitative assessment of ventricular function in the practice of clinical cardiology^1^.

Recently with the development of parallel imaging and compressed sensing^5–7^, real-time MRI has been demonstrated to be feasible^8–13^. It has also been evidenced in cardiology that this technique can provide volumetric measurements comparable to those with retrospective cine^14^. Although real-time cardiac MRI gives worse image quality than retrospective cine due to the use of data undersampling for imaging acceleration, it offers several advantages including less logistical challenges associated with breath-holding and ECG gating^15^, more reliable images in arrhythmia patients^16^, and the ability to investigate cardio-respiratory coupling^17^. In addition, real-time images are more informative because they permit visualization of the temporal and spatial behaviors of cardiac motion over a series of sequential cardiac cycles. This multi-cycle temporospatial information, although potentially beneficial to ventricular function assessment, has not been investigated extensively in cardiac MRI studies.

The presented work was therefore to develop a real-time cardiac MRI approach to temporospatial characterization of ventricular wall motion for quantitative assessment of ventricular function. This approach sought to image a series of sequential cardiac cycles instead of a single cardiac cycle like in conventional retrospective cine for volumetric measurements. By characterizing time-series signals related to ventricular wall motion with Fourier transform^18^ and correlation analysis^19^, two temporospatial indices, temporal periodicity and spatial coherence, were calculated to evaluate ventricular performance. For proof-of-concept, a cardiac MRI study was conducted with healthy volunteers and heart failure (HF) patients. Temporospatial characterization with real-time cardiac MRI was investigated prospectively in reference to conventional volumetric measurements with retrospective cine. This study was to demonstrate that temporal periodicity and spatial coherence would offer the ability to detect the difference of ventricular performance between the healthy volunteers and HF patients.

## Results

### Subjects and images

A total of 12 healthy volunteers and 12 HF patients were recruited in the presented work. The healthy volunteers included 7 females and 5 males with ages ranged from 22 to 72 years (average age 56 years). The HF patients included 4 females and 8 males with ages ranged from 29 to 74 years (average age 51 years). These patients had reduced systolic function either in the left ventricle (LV) or in the right ventricle (RV). The MRI assessment results from cardiologists are provided by Table 1. Also presented in the table is a list of diagnosed cardiovascular and pulmonary diseases in every HF patient.

**Table 1:** Diagnosed diseases and cardiac MRI assessment of the recruited HF patients.

Patient ID	Diagnosed diseases		Cardiac MRI assessment			
			LV		RV	
	Type of HF	Other cardiovascular and pulmonary problems	Size	Systolic function	Size	Systolic function
1	Chronic systolic	Nonischemic cardiomyopathy, shortness of breath, palpitation	Severely dilated, EDV index = 154 ml/m^2^	Moderately reduced, EF = 37%	Normal, EDV index = 90 ml/m^2^	Normal, EF = 56%
2	Chronic systolic	Hypertension, abnormal stress test	Moderately dilated, EDV index = 110 ml/m^2^	Moderately reduced, EF = 42%	Normal, EDV index = 92 ml/m^2^	Normal, EF = 57%
3	Acute systolic	Atrial fibrillation, stroke, cardiomyopathy, palpitation	Normal EDV index = 90 ml/m^2^	Severely reduced, EF = 33%	Normal, EDV index = 76 ml/m^2^	Mildly reduced, EF = 41%
4	Chronic diastolic	Hypertension, pulmonary hypertension, atrial fibrillation	Mildly dilated, EDV index = 104 ml/m^2^	Normal, EF = 57%	Severely dilated, EDV index = 141 ml/m^2^	Mildly reduced, EF = 42%
5	Chronic systolic	Hypertension, cardiomyopathy, chronic obstructive pulmonary disease	Normal, EDV index = 73 ml/m^2^	Severely reduced, EF = 27%	Normal, EDV index = 72 ml/m^2^	Moderately reduced, EF = 37%
6	Chronic systolic	Cardiomyopathy, paroxysmal atrial fibrillation	Normal, EDV index = 81 ml/m^2^	Moderately reduced, EF = 39%	Normal, EDV index = 82 ml/m^2^	Mildly reduced, EF = 43%
7	Acute on chronic systolic	Atrial fibrillation, stroke, nonischemic cardiomyopathy, transient ischemic attack, chest pain, palpitation	Normal, EDV index = 90 ml/m^2^	Severely reduced, EF = 32%	Normal, EDV index = 90 ml/m^2^	Mildly reduced, EF = 40%
8	Chronic diastolic	Nonischemic cardiomyopathy, atrial fibrillation	Normal, EDV index = 74 ml/m^2^	Mildly reduced, EF = 46%	Normal, EDV index = 55 ml/m^2^	Normal, EF = 51%
9	Acute on chronic systolic	Shortness of breath	Severely dilated, EDV index = 160 ml/m^2^	Severely reduced, EF = 31%	Normal, EDV index = 90 ml/m^2^	Moderately reduced, EF = 32%
10	Acute systolic	Restrictive cardiomyopathy, dyspnea on exertion, mitral valve prolapses, hypertension	Moderately dilated, EDV index = 114 ml/m^2^	Moderately reduced, EF = 40%	Normal, EDV index = 67 ml/m^2^	Normal, EF = 56%
11	Chronic diastolic	Pulmonary embolism, arteriosclerotic heart disease	Normal, EDV index = 72 ml/m^2^	Normal, EF = 50%	Normal, EDV index = 66 ml/m^2^	Mildly reduced, EF = 49%
12	Chronic systolic and diastolic	Premature ventricular contraction, atrioventricular block, left anterior fascicular block, hypertension, coronary artery disease, chest pain	Normal, EDV index = 98 ml/m^2^	Moderately reduced, EF = 41%	Normal, EDV index = 67 ml/m^2^	Normal, EF = 58%

**Figure 1:**
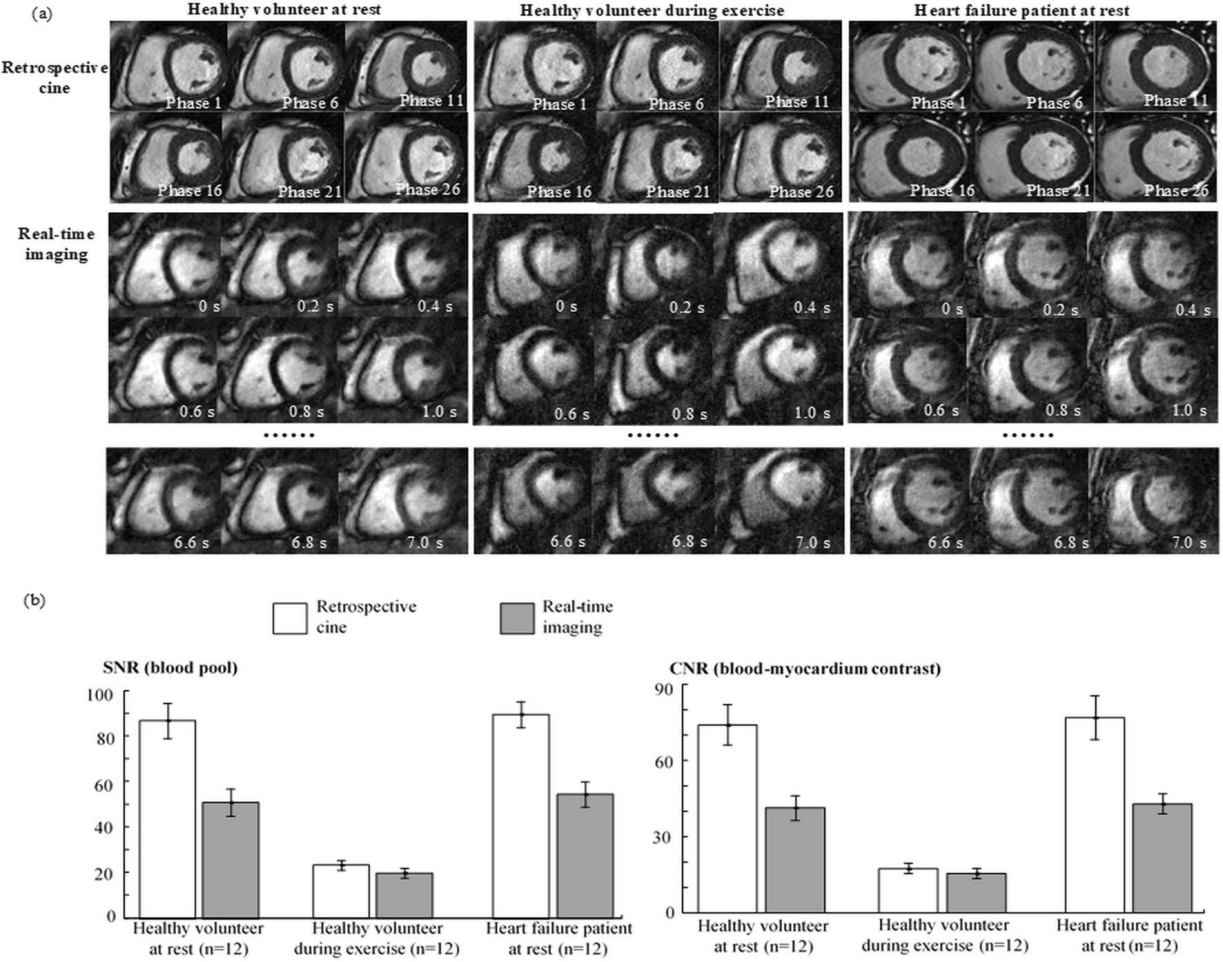
(**a**) Selected time frames of the image examples from retrospective cine and real-time imaging in a resting-state healthy volunteer, an exercising-state volunteer, and a heart failure patient. (**b**) Bar plots of SNR and CNR measurements in retrospective cine and real-time images from different groups of the subjects. Each bar is presented as mean ± standard deviation (n = 12).

The healthy volunteers were scanned both at rest and during exercise. The HF patients were scanned only at rest. Figure 1(a) provides examples of the retrospective cine and real-time images collected respectively from a resting-state healthy volunteer, an exercising-state healthy volunteer, and a resting-state HF patient. In comparison to retrospective cine, real-time imaging gave more noise and artifacts. However, real-time images permitted visualization of systolic contraction and diastolic relaxation overs a series of sequential cardiac cycles, providing more temporospatial information about cardiac motion. Figure 1(b) shows the bar plots for blood-pool signal to noise ratio (SNR) and blood-myocardium contrast to noise ratio (CNR) measured in different subject groups. At rest, real-time imaging gave ~ 50% lower SNR and CNR than those given by retrospective cine. During exercise, retrospective cine showed more loss of image quality than real-time imaging. The SNR and CNR of real-time images were thus only slightly lower than those of retrospective cine images. This finding agreed with a previous study on real-time exercise stress cardiac MRI^12^: Because heartbeats would be unstable during exercise, there was considerable inconsistency in retrospective data collected from different cardiac cycles. As a result, retrospective cine suffered more performance degradation during exercise than real-time imaging.

### Volumetric measurements

Volumetric indices, including end-diastole volume (EDV), end-systole volume (ESV), stroke volume (SV), and ejection fraction (EF), were measured with retrospective cine and real-time images in the LV and RV. The volumetric measurements with retrospective cine images provided a clinical standard for quantitative assessment of cardiac function. This clinical standard served two purposes: First, a comparison between the volumetric measurements with real-time images and the clinical standard provided validation on whether real-time images offered sufficient quality for cardiac MRI quantification. Second, the clinical standard provided a reference for evaluating the ability of temporospatial characterization to quantitatively assess ventricular function in HF patients. In volumetric measurements, the normality of data distribution was confirmed with in each subject group an Anderson–Darling test (n = 12; *P* > 0.2). A t-test was used to compare volumetric index measurements in different subject groups.

Table 2 provides a summary of volumetric measurements with retrospective cine and real-time images. Two measurements were comparable. They were also comparable to those in previous studies^12,14,20–22^. It was noticed that the measurements with real-time images gave smaller standard deviation than those with retrospective cine, especially during exercise. This agreed with a previous study on real-time exercise cardiac MRI^12^. We calculated the differences of EDV, ESV, SV and EF measurements with real-time images and those with retrospective cine images. These differences were comparable to those in a previous study on volumetric measurements with real-time imaging^14^.

**Table 2:** Summary (mean ± standard deviation) of the volumetric measurements with retrospective cine (RC) and real-time (RT) images and the measurement differences (DIFF) between two methods.

	Resting-state healthy volunteers (n = 12)			Exercising-state healthy volunteers (n = 12)			Resting-state HF patients (n = 12)		
	RC	RT	DIFF	RC	RT	DIFF	RC	RT	DIFF
LV-EDV (ml)	149 ± 31	143 ± 28	− 6.2 ± 5.7	148 ± 36	142 ± 27	− 5.8 ± 10	213 ± 53	207 ± 48	− 5.8 ± 7.9
LV-ESV (ml)	67 ± 17	59 ± 13	− 7.8 ± 8.1	59 ± 22	50 ± 16	− 8.3 ± 12	129 ± 40	122 ± 35	− 6.1 ± 7.5
LV-SV (ml)	83 ± 16	84 ± 16	1.6 ± 5.8	90 ± 21	92 ± 13	2.4 ± 11	84 ± 25	84 ± 21	0.33 ± 6.7
LV-EF (%)	56 ± 4.2	59 ± 2.7	3.4 ± 4.4	61 ± 9.3	65 ± 5.3	4.0 ± 6.4	40 ± 8.4	41 ± 6.2	1.2 ± 3.2
RV-EDV (ml)	137 ± 32	130 ± 24	− 6.8 ± 7.9	139 ± 42	134 ± 28	− 4.6 ± 15	174 ± 62	171 ± 57	− 3.0 ± 6.7
RV-ESV (ml)	53 ± 19	46 ± 12	− 7.0 ± 7.4	46 ± 18	41 ± 14	− 5.8 ± 10	94 ± 42	88 ± 39	− 6.1 ± 3.6
RV-SV (ml)	84 ± 24	84 ± 18	0.25 ± 7.4	93 ± 29	94 ± 17	− 1.2 ± 16	80 ± 26	83 ± 23	3.1 ± 4.5
RV-EF (%)	61 ± 9.7	64 ± 6.4	3.3 ± 4.7	67 ± 7.4	70 ± 5.2	3.4 ± 7.5	47 ± 8.7	50 ± 8.2	2.8 ± 1.7

**Figure 2:**
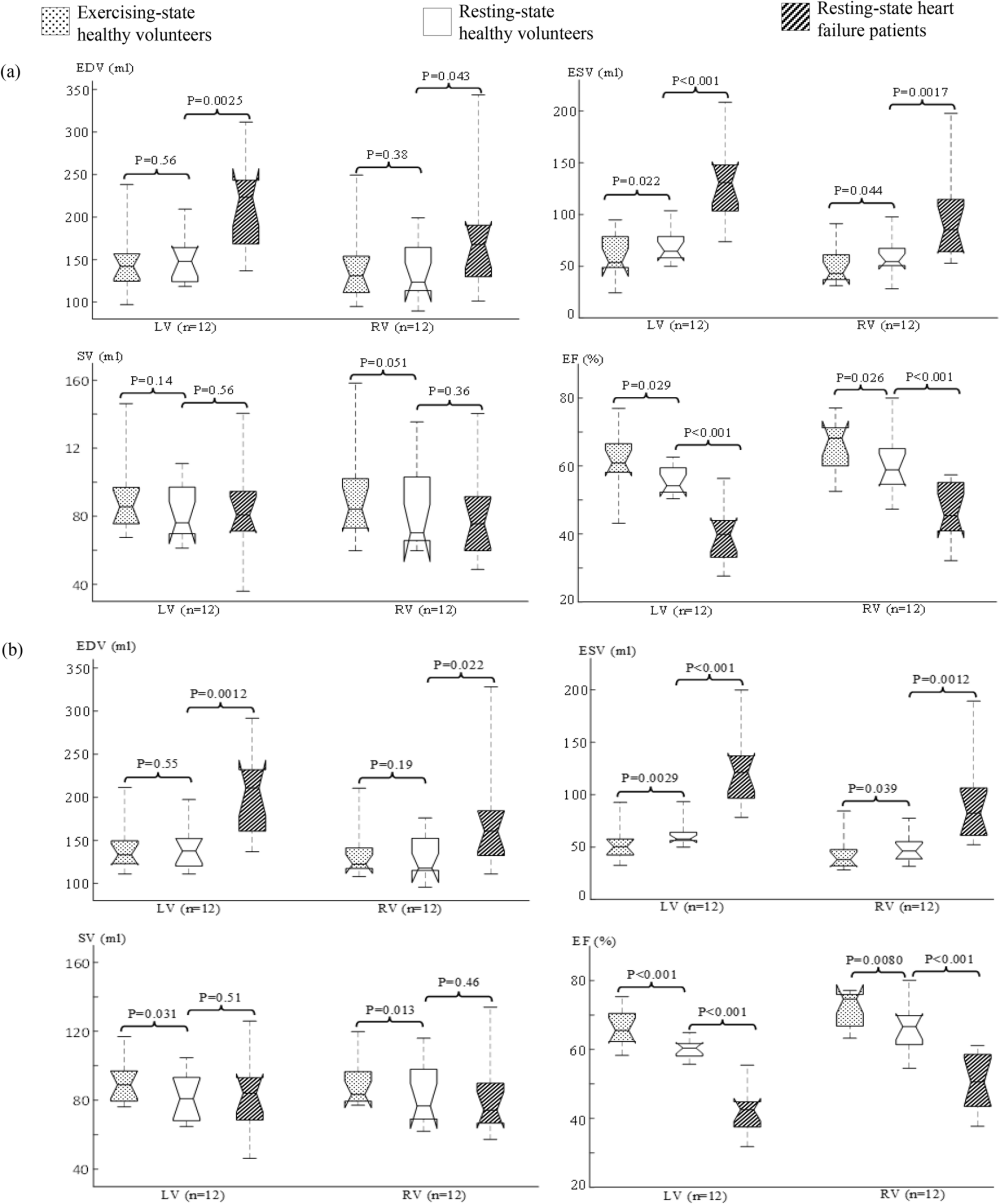
Box plots of the volumetric measurements with retrospective cine (**a**) and real-time imaging (**b**) in the healthy volunteers and HF patients. Each box provides the median, 25th and 75th percentiles of the measurements and the numbers give the *t*-test *P* values (n = 12).

Figure 2(a) provides the t-test results with retrospective cine images. In the healthy volunteers, exercise induced a significant decrease of ESV (n = 12; *P* = 0.022 for LV and *P* = 0.044 for RV) and a significant increase of EF (n = 12; *P* = 0.029 for LV and *P* = 0.026 for RV). No significant differences were found between the resting- and exercising-state measurements of EDV (n = 12; *P* = 0.56 for LV and *P* = 0.38 for RV) and SV (n = 12; *P* = 0.14 for LV and *P* = 0.051 for RV). In comparison to the healthy volunteers, the HF patients presented a higher EDV (n = 12; *P* = 0.0025 for LV and *P* = 0.043 for RV), a higher ESV (n = 12; *P* < 0.001 for LV and *P* = 0.0017 for RV), and a lower EF (n = 12; *P* < 0.001 for both LV and RV). No significant differences were found in the SV measurements between the healthy volunteers and the HF patients (n = 12; *P* = 0.56 for LV and *P* = 0.36 for RV).

Figure 2(b) provides the t-test results with real-time images. Most results were comparable to those with retrospective cine images (Fig. 2a): The healthy volunteers presented a decrease of ESV and an increase of EF during exercise in comparison to those at rest. The HF patients gave a higher EDV, a higher ESV, and a lower EF than those in the healthy volunteers. There were no significant differences in EDV measurements between the resting- and the exercising-state healthy volunteers, and in SV measurements between the healthy volunteers and the HF patients. However, real-time imaging detected an increase of SV due to exercise in the healthy volunteers (n = 12; *P* = 0.031 for LV and *P* = 0.012 for RV) while retrospective cine could not. This performance difference, as suggested in a previous study^12^, was because real-time imaging gave a smaller standard deviation in volumetric measurements than that given by retrospective cine, especially during exercise (Table 2).

### Measurements of temporal periodicity and spatial coherence in real-time images

**Figure 3:**
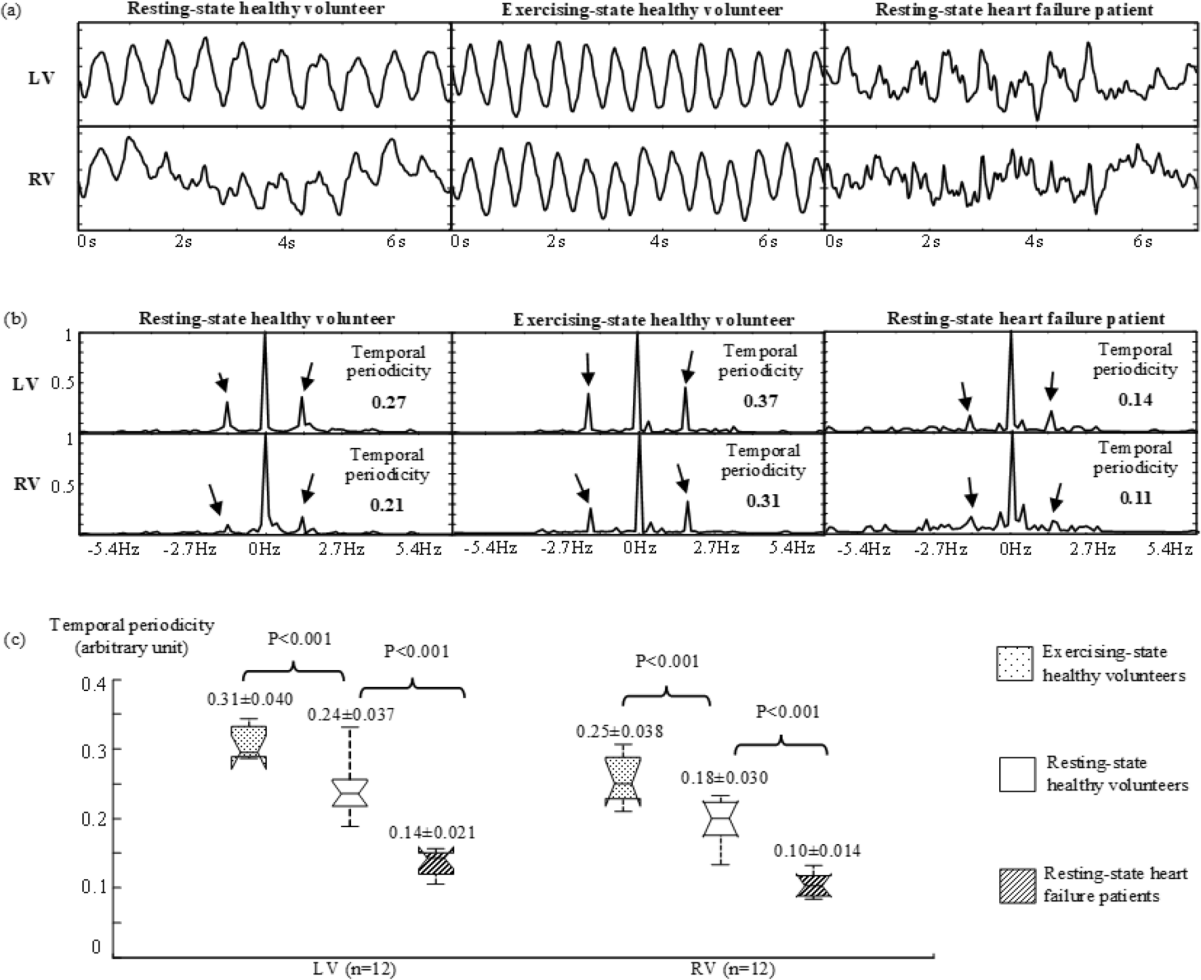
(**a**) Examples of reference wall-motion signals within LV-ROI (top row), and RV-ROI (bottom row). The unit on vertical axes is arbitrary. (**b**) Fourier transforms of the reference wall-motion signals in (**a**). The spectral magnitudes are normalized with respect to the zero-frequency component. The arrows indicate the cardiac frequency components for measuring temporal periodicity. (**c**) Box plots of the temporal periodicity measurements in real-time images from the healthy volunteers and HF patients. Each box provides the median, 25th and 75th percentiles of the measurements and the numbers give the mean ± standard deviation, and the *t*-test *P* values (n = 12).

In every subject, a region of interest (ROI) was defined for the ventricle (LV or RV). Within each ROI (LV-ROI or RV-ROI), a reference wall-motion signal was generated for calculating temporal periodicity with Fourier transform^18^ and spatial coherence with correlation analysis^19^ (details in Methods). Figure 3(a) provides examples of the reference wall-motion signals, respectively, for LV and for RV in the same subjects as in Fig. 1(a). All the reference wall-motion signals showed a periodic alternation of increment and decrement associated with ventricular contraction and relaxation over a series of sequential cardiac cycles. In addition, noise-like temporal fluctuation was present over all the cardiac cycles, resulting in considerable aperiodicity. In comparison, the HF patient exhibited the highest aperiodicity, and the exercising-state healthy volunteer the lowest. Corresponding to different aperiodicity in time domain, the reference wall-motion signals gave different cardiac frequency components in frequency domain (Fig. 3b): The exercising-state healthy volunteer presented the strongest cardiac frequency components and the HF patient the weakest (marked by arrows).

The normality of temporal periodicity measurements was confirmed in each subject group with an Anderson–Darling test (n = 12; *P* > 0.5). Figure 3(c) provides the t-test results for the comparison between different subject groups. The measurements of temporal periodicity were found to be significantly different: In comparison to those in the resting-state healthy volunteers (0.24 ± 0.037 for LV and 0.18 ± 0.030 for RV, in arbitrary unit), they were higher in the exercising-state healthy volunteers (0.31 ± 0.040 for LV and 0.25 ± 0.038 for RV, in arbitrary unit; n = 12; *P* < 0.001) and lower in the resting-state HF patients (0.14 ± 0.021 for LV and 0.10 ± 0.014 for RV, in arbitrary unit; n = 12; *P* < 0.001).

**Figure 4:**
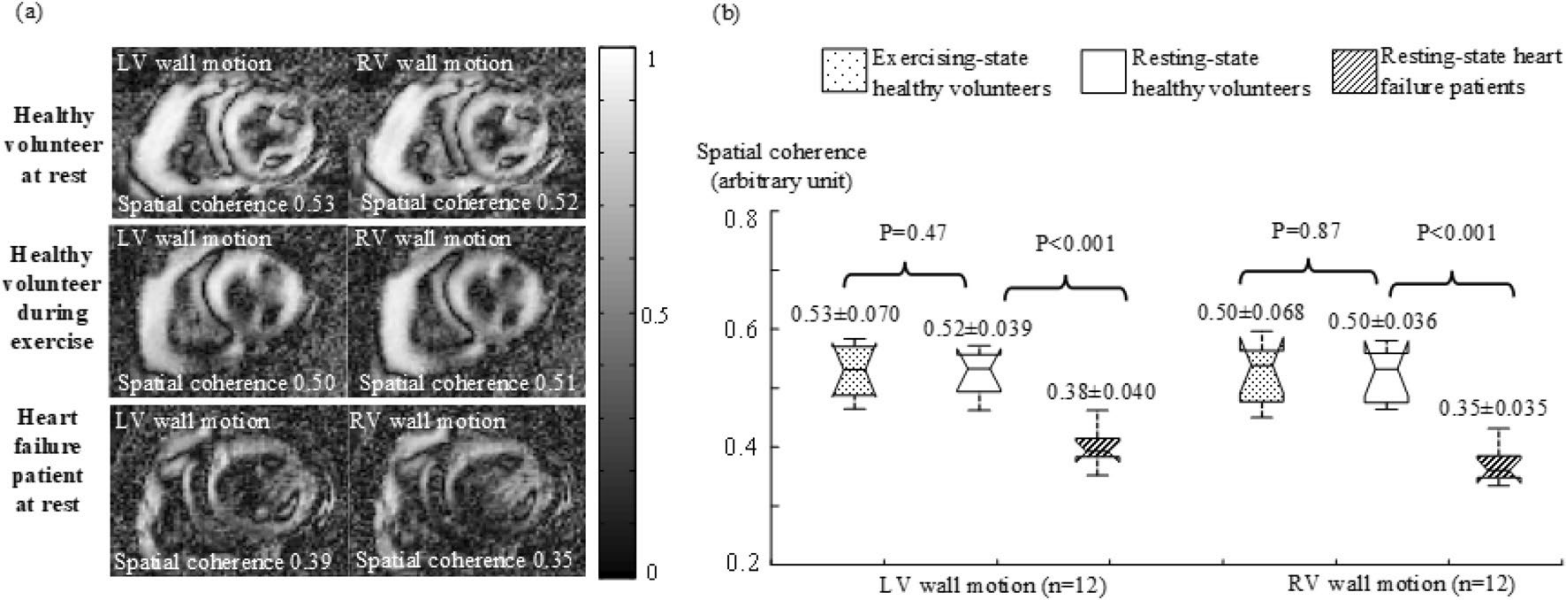
(**a**) Examples of correlation maps with spatial coherence measurements for LV and RV wall motion. (**b**) Box plots of spatial coherence measurements in real-time images from the healthy volunteers and HF patients. Each box provides the median, 25th and 75th percentiles of the measurements and the numbers give the mean ± standard deviation, and the *t*-test *P* values (n = 12).

Correlation analysis^19^ was performed between the reference wall motion signal within each ROI and every time-series signal at different spatial locations (details in Methods), generating two correlation maps respectively for LV wall motion and RV wall motion. The spatial coherence was calculated from each correlation map for LV or RV wall motion. Figure 4(a) provides examples of the correlation maps measured in the same subjects as in Fig. 1(a). The correlation was found to be globally higher in the healthy volunteer than that in the HF patient. Correspondingly, the HF patient gave lower spatial coherence. In each subject group, the normality of spatial coherence measurements was confirmed with an Anderson–Darling test (n = 12; *P* > 0.3). Figure 4(b) provides the t-test results for the comparison between different subject groups. The measurements of spatial coherence were found to be comparable during exercise and at rest in the healthy volunteers (0.53 ± 0.070 vs. 0.52 ± 0.039 for LV wall motion and 0.50 ± 0.068 vs. 0.50 ± 0.036 for RV wall motion, in arbitrary unit; n = 12; *P* > 0.4). In contrast, the HF patients presented significantly lower spatial coherence (0.38 ± 0.040 for LV wall motion and 0.35 ± 0.035 for RV wall motion, in arbitrary unit; n = 12; *P* < 0.001).

### Comparison of temporospatial and volumetric indices

**Figure 5:**
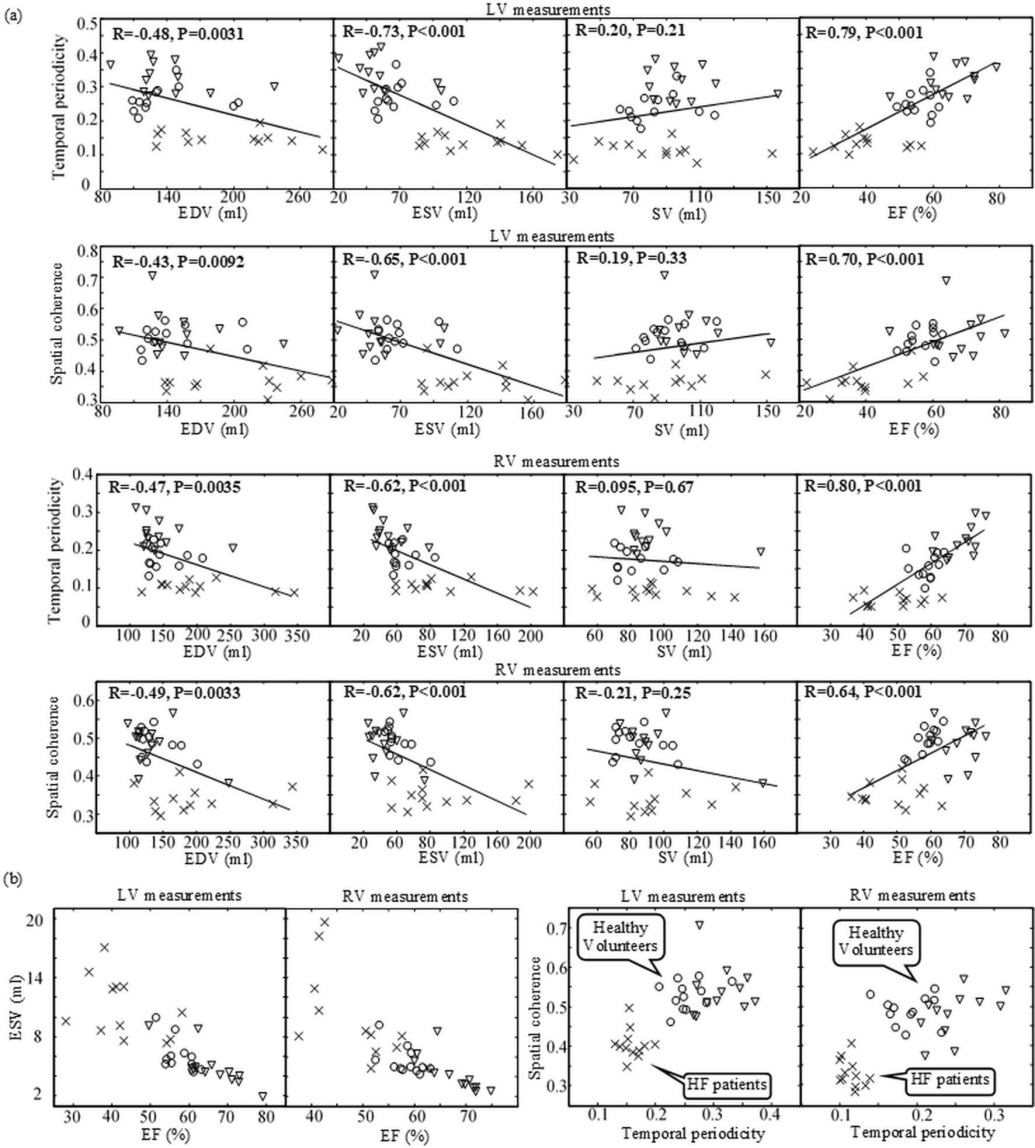
(**a**) Pearson correlation coefficients between temporospatial indices (temporal periodicity and spatial coherence) and volumetric indices (EDV, ESV, SV and EF) in LV and RV. (**b**) Scatter plots of volumetric indices (ESV vs. EF) and those of temporospatial indices (spatial coherence vs. temporal periodicity). The measurements in the healthy volunteers at rest and during exercise are represented respectively with “o” and “∇”, and those in the HF patients with “× ”.

Figure 5(a) provides the Pearson correlation coefficients between the temporospatial indices (temporal periodicity and spatial coherence) and the volumetric indices (EDV, ESV, SV and EF). The temporospatial indices were most correlated to EF (R = 0.64–0.80). They were strongly correlated to ESV (R = 0.62–0.73). Their correlation with EDV, although not high, exists (R = 0.43–0.49). There was no considerable correlation with SV in all the cases (R < 0.30). Figure 5(b) provides the scatter plots of spatial coherence vs. temporal periodicity in reference to those of ESV vs. EF. The HF patients and healthy volunteers were clearly separated in the scatter plots of temporospatial indices while they were mixed in those of volumetric indices.

We recorded the post-processing time for measuring temporal periodicity and spatial coherence in real-time images. The most time-consuming procedure was image segmentation for ROI determination in the LV and RV. This manual procedure required 10–20 min for each subject depending on the quality of real-time images. After ROI determination, the time cost of automatic index computation was ~ 2 s on a computer with Intel (R) Core (TM) i7-6700 CPU 3.40 GHz and 32 GB memory. In comparison, volumetric measurements with retrospective cine required 5–10 min for image segmentation and ~ 0.5 s for automatic index computation on the same computer.

## Discussion

The presented work proposed a real-time cardiac MRI approach to measuring temporal periodicity and spatial coherence of LV and RV wall motion in a series of sequential cardiac cycles. It was experimentally found that the temporospatial indices detected the difference of ventricular performance between the healthy volunteers and the HF patients. This demonstrated the potential of temporospatial characterization with real-time cardiac MRI for quantitative assessment of ventricular function.

Intuitively, ventricular wall motion may be characterized by tracking ventricular wall anatomy spatially along the time with real-time images. Practically, the direct tracking is difficult because the ventricular walls give low SNR in the images. For this reason, temporospatial characterization seeks to identify the high-SNR blood pool regions, and define two ROIs (LV-ROI and RV-ROI) that give an estimate of the spatial range of ventricular wall motion in every cardiac cycle. The MRI signals within an ROI arise primarily from the ventricular walls. In addition, they can be affected by the surrounding tissues such as pericardium, lumen and blood that may move into the ROI with systolic contraction and diastolic relaxation. However, as these surrounding tissues always move together with the ventricular walls, the dynamic changes of MRI signals within the ROI are dependent primarily on ventricular wall motion. The ROI-based temporospatial characterization thus provides an indirect approach to analyzing ventricular wall motion with real-time cardiac MRI.

**Figure 6:**
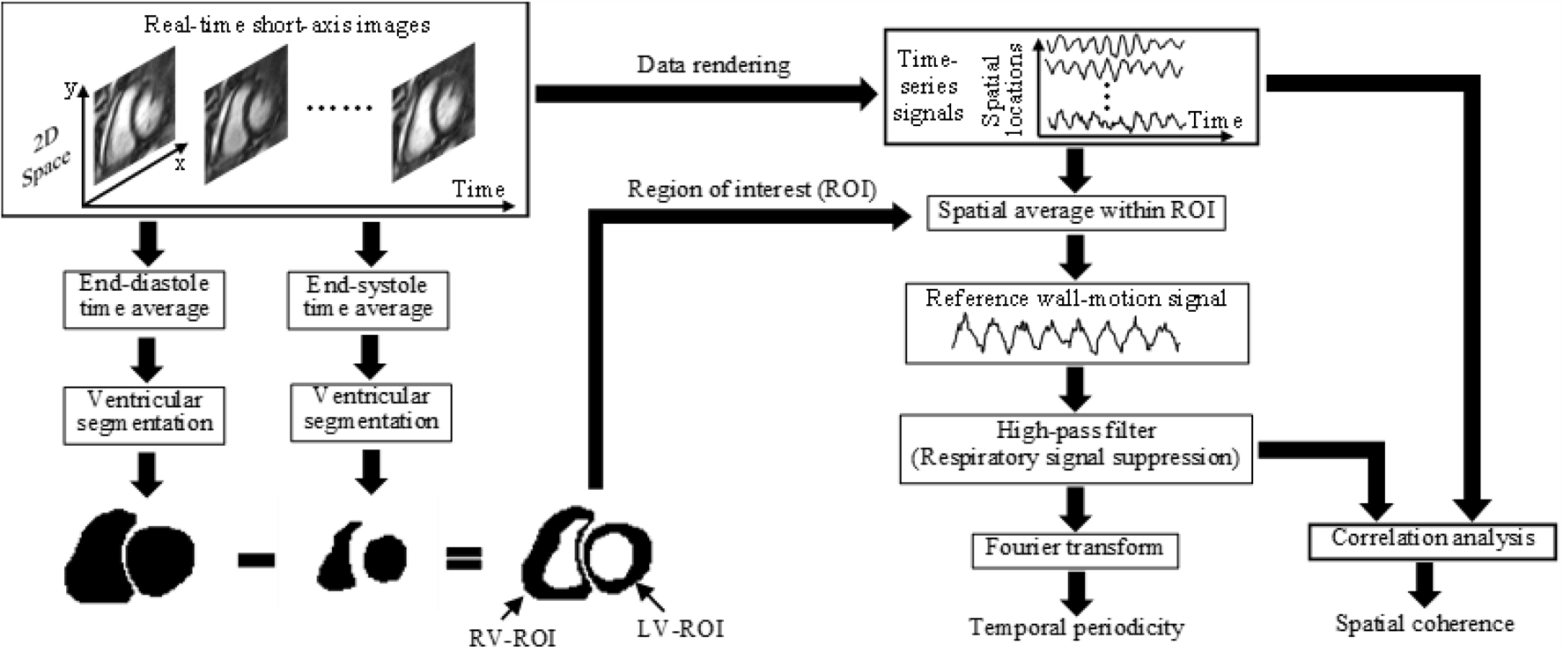
A diagram for temporospatial characterization with real-time cardiac MRI.

Temporospatial characterization relies on the calculation of a reference wall-motion signal from spatial averaging in each ROI (Fig. 6). The rationales for spatial averaging are twofold: First, spatial averaging improves SNR performance in real-time time-series, providing a robust approach to locating the spectral peak associated with the heartbeats in the Fourier transform for measuring temporal periodicity. Second, it is known that ventricular motion spreads spatially through the ventricular walls during every heartbeat^23,24^, allowing the ventricular wall motion to be synchronized at different spatial locations. Due to the synchronization, there should exist a common motion pattern along the ventricular walls. This common motion pattern can affect the dynamic changes of MRI signal at every spatial location and may be estimated from the spatial averaging of time-series signals. Accordingly, the reference wall-motion signal, which is calculated by spatially averaging the time-series signals within an ROI, gives an estimate of the common pattern of ventricular wall motion in LV or RV. This estimate can be used as a reference in correlation analysis for measuring how well ventricular wall motion is synchronized at different spatial locations, i.e., spatial coherence. As the measurements of temporal periodicity and spatial coherence are both based on the reference wall-motion signal arising from spatially averaged ventricular wall motion, they provide quantitative assessment of global ventricular function.

Temporal periodicity is an engineering concept for analyzing time-series signals^18^. If a time-series signal is periodic, its Fourier transform has a non-zero magnitude only at the frequency corresponding to its temporal period. In this ideal case, the temporal periodicity (Eq. 1 in Methods) is equal to one. A practical time-series signal always has aperiodicity. In a reference wall-motion signal that arises from ventricular wall motion, we have noticed the following aperiodic behaviors. First, ventricular wall motion is driven by heartbeats that naturally have variability^25,26^. Second, myocardial contraction and relaxation may occasionally present a certain temporal inconsistency within a cardiac cycle or across different cardiac cycles. Due to these aperiodic behaviors, the reference wall-motion signal has noise-like temporal fluctuation (Fig. 3a). Its Fourier transform correspondingly shows a "leakage" of the energy from a cardiac frequency to other frequencies (Fig. 3b). This spectral "leakage" may lower the temporal periodicity.

We believe that, to maintain a consistent cardiac output, a healthy heart should keep the temporal periodicity at a certain level in the LV and RV wall motion. This temporal periodicity, which can be measured from the reference wall-motion signal, is expected to be higher when there is a need for more cardiac output, e.g., during exercise. On the other hand, if there is a loss of ventricular function due to heart diseases, the aperiodic patterns may become stronger, resulting in lower temporal periodicity in the reference wall-motion signal. As demonstrated in our study (Fig. 3), the reference wall-motion signal in a healthy volunteer showed less noise-like temporal fluctuation and stronger periodic patterns during exercise than that at rest. In comparison, a HF patient presented stronger noise-like temporal fluctuation over all the cardiac cycles in the reference wall-motion signal. As a result, temporal periodicity was higher in the exercising-state healthy volunteers, but lower in the resting-state HF patients, in comparison to that in the resting-state healthy volunteers. These findings validate the physiological relevance of temporal periodicity.

Spatial coherence (Eq. 2 in Methods) evaluates the spatial spread of ventricular wall motion over a series of sequential cardiac cycles. Motion may spread along the ventricular walls^23,24^, and also from the ventricular walls to the surrounding tissues inside or outside the ventricle during contraction and relaxation. We believe that ventricular performance should be dependent on how widely ventricular wall motion would spread over the entire ventricular anatomy. In the presented work, we measured the correlation between the reference wall-motion signal and every real-time cardiac MRI signal at different spatial locations in the ventricular anatomy. A correlation map was generated to investigate the spatial spread of the LV or RV wall motion. For a global assessment, spatial coherence was calculated by averaging a correlation map within the ventricular anatomy. As shown in Fig. 4, the correlation maps indicated that the LV and RV wall motion spread less widely over the ventricular anatomy in the HF patients than that in the healthy volunteers. Correspondingly, the HF patients gave lower spatial coherence than that in the healthy volunteers. These findings suggest that the reduced systolic function should be attributed to less spatial spread of the ventricular wall motion in addition to temporal aperiodicity, and abnormal myocardium may suffer from the impediment of motion spread.

We have found that temporal periodicity and spatial coherence are correlated strongly (R > 0.5) with EF and ESV, moderately (0.5 > R > 0.3) with EDV, and weakly (R < 0.3) with SV (Fig. 5a). These findings further evidence the physiological relevance of temporospatial characterization with real-time cardiac MRI. Especially, the temporospatial indices were found to be most correlated to EF, indicating that they should be related to both systolic contraction and diastolic preload in a cardiac cycle. This strong correlation may be partially explained by the fact that both temporospatial indices and EF are normalized. Because of the normalization, temporospatial indices provide inter-subject comparability.

Despite the correlation, temporospatial characterization and volumetric measurements extract different information from cardiac MRI. Volumetric indices give an estimate of the change of blood volume from the end of diastole to that of systole in a cardiac cycle and are thereby insensitive to dynamic events in the midst of ventricular filling and ejection. In contrast, as Fourier transform and correlation analysis are dependent on the data measurements at every time point, temporospatial indices characterize the ventricular wall motion temporally and spatially throughout the systole and diastole over a series of sequential cardiac cycles. With more information, temporospatial indices can provide better assessment of the difference of ventricular performance between the healthy volunteers and the HF patients than that given by volumetric measurements. This explains why the HF patients and healthy volunteers are clearly separated in the scatter plots of spatial coherence against temporal periodicity while they are mixed in those of ESV against EF (Fig. 5b). Our findings suggest that spatial coherence and temporal periodicity may provide the metrics of ventricular function for detection of the change of myocardial performance that is independent of ventricular size. Therefore, temporospatial characterization with real-time cardiac MRI has the potential to be complementary to the traditional volumetric measurements with retrospective cine.

It should be noted that Table 1 includes three HF patients (Patient ID = 4, 8 and 11) with only diastolic dysfunction. In these patients, the systolic function of one ventricle was mildly reduced and that of the other was normal. They were found among those HF patients mixed with the healthy volunteers in the scatter plots of ESV against EF (Fig. 5b, left-side). This may be related to the poor performance of volumetric measurements in quantitative assessment of diastolic dysfunction , as demonstrated in previous studies^27,28^. From the scatter plots of spatial coherence against temporal periodicity (Fig. 5b, right-side), it is likely that the temporospatial indices be responsive to the diastolic dysfunction in HF patients. Although the evidence is not strong here due to the small number of such patients, this implies that the significance of a further study on temporospatial indices would be high. It should also be noted that Table 1 includes five HF patients (Patient ID = 2, 4, 9, 11 and 12) without any diseases that could cause observable ventricular wall motion abnormalities (e.g., cardiomyopathies). This indicates that temporospatial characterization of ventricular wall motion offers the sensitivity to detect ventricular dysfunction that cannot be identified directly by reviewing the images.

It is ideal to assess ventricular function by temporospatial characterization of 3D ventricular wall motion. However, 3D motion analysis is technically limited by the availability of 3D real-time imaging. With available 2D real-time imaging, a reference wall-motion signal may be generated which extracts the information mostly about ventricular wall motion along the radial or the circumferential direction on the short-axis plane. Although the short-axis MRI signal is also dependent on through-plane wall motion along the longitudinal direction, this dependence varies from the basal to the apical slices due to the non-uniform longitudinal movement during contraction^29^. In the presented work, the healthy volunteers and HF patients were scanned and compared with the same 2D short-axis slice coverage. This experimental strategy minimized the statistical bias related to motion difference between the basal and the apical slices within every subject. However, the future work should address how the underestimated longitudinal motion would affect temporospatial indices, especially in the assessment of RV function that may be dependent strongly on longitudinal contraction^29^.

The presented work is a proof-of-concept study on temporospatial characterization of ventricular wall motion with real-time cardiac MRI. As ventricular wall motion is the cause of many mechanic events in a cardiac cycle, such as pressure, volume and flow velocity changes as well as the response to the change of electrical conduction^30^, this characterization may provide information about the intricacy of wall motion in normal subjects and in patients with a number of different cardiac diseases. Accordingly, a small group of HF patients is not sufficient for a comprehensive evaluation of the clinical potential of temporospatial indices. The follow-up studies should expand into an experimental work on a wider range of cardiac diseases with a larger number of human subjects. Additional future work should also address inter-operator, intra-operator, and inter-exam reproducibility; comparison with other LV functional parameters and techniques; and how to improve real-time image quality.

Given their fully quantitative nature, temporal periodicity and spatial coherence have the potential to detect an early change of myocardial performance before the reduction of global systolic function. Potential applications include quantitative assessment of LV function during stress (exercise or pharmacological) MRI; measurement of left atrial functional performance parameters; assessment of LV diastolic function; and assessment of the RV function in various disease states (including pulmonary hypertension and congenital heart disease). They may also have the potential to allow effective assessment of therapeutic response in patients with cardiomyopathy.

In conclusion, real-time cardiac MRI enables an approach to characterizing temporospatial behaviors of ventricular wall motion with two quantitative indices, temporal periodicity and spatial coherence, which may provide quantitative assessment of the LV and RV function beyond conventional volumetric measurements with retrospective cine.

## Methods

### Experimental study

A cardiac MRI study with healthy volunteers and HF patients was conducted under the approval of the Institutional Review Board (St. Francis Hospital Cardiac Imaging Research Protocol Review Committee: approval number 18-03). The study followed the principles of the Declaration of Helsinki and the regulations of the St. Francis Hospital human ethic committee. Informed consent and medical history questionnaires were obtained from all the subjects. The study exclusions included metallic hazards, pacemaker/defibrillator device, claustrophobia and conditions not appropriate for exercise. In addition, the subjects with a resting heart rate greater than 90 bpm or a systolic blood pressure greater than 180 mmHg were excluded.

A healthy volunteer was recruited if there was no history of cardiovascular disease and no major risk factor. In addition, the 12-lead ECG and echocardiography examination were normal. A HF patient was recruited if there was a history of hospitalized heart failure. The healthy volunteers were scanned at rest and during exercise. The HF patients were scanned at rest only. All the scans were run with a balanced steady state free precession sequence and a 12-channel coil array on a 1.5 T clinical scanner (Siemens Healthineers, Erlangen, Germany). In the exercising-state scans, an MRI-compatible exercise supine bicycle (Lode B.V., Groningen, the Netherlands) was mounted on the scanner table. The imaging data were acquired while a volunteer was exercising inside the scanner. During exercise, the volunteer's heart rate and rhythm, and oxygen saturation were monitored continuously. The blood pressure was measured every 3 min. The intensity of exercise was adjusted by the bike resistance, which was increased by 25 Watts every 3 min until the heart rate was consistently in the range of 100–110 bpm.

Retrospective cine and real-time imaging data were both collected. The ventricular anatomy was covered by 10 short-axis slices. The retrospective cine scan was run with breath-holding and ECG gating. The real-time imaging scan was run using radial sampling during free-breathing and without ECG gating. In addition, the following acquisition parameters were used:

- ECG-gated retrospective cine: FOV 340 × (220–250) mm, voxel 1.5–1.9 mm, segments 5–8, iPAT factor 2, ECG-synchronized phases 30, TR/TE 2.6/1.3 ms, FA 50°–75°, slice thickness 8 mm, slice gap 2 mm, bandwidth 1420 Hz.
- Real-time imaging with radial sampling: FOV 230–250 mm, voxel 1.5–1.9 mm, TR/TE 2.2–3.0/1.1–1.5 ms, FA 50°–75°, slice thickness 8 mm, slice gap 2 mm, bandwidth 1510 Hz, time frames 384, a total of 3072 radial views.

### Image reconstruction

Retrospective cine images were reconstructed online with the system software provided by the MRI manufacturer. Each series of the retrospective images included 30 phases in a single cardiac cycle, providing a temporal resolution of 30–40 ms. Real-time images were reconstructed from the raw data offline in MATLAB (The MathWorks, Inc., Massachusetts, United States). The reconstruction was performed with a lab-developed algorithm based on the real-time imaging works previously published by several different research groups^8,9,11–13^. Each series of the real-time images included 7–16 cardiac cycles with a temporal resolution of 18–30 ms in a time window of ~ 7 s. There were 33–48 phases in every cardiac cycle at rest and 24–32 phases during exercise.

### Post-processing

In post-processing, the retrospective cine images were analyzed with a semi-automatic segmentation method^31^ that sought to delineate the LV and RV borders based on both image contrast between the blood pool and the myocardium and prior information about ventricular geometry and structures. The segmented end-diastole and end-systole images for all the short-axis slices were used to measure LV and RV volumes as in previous studies^32–34^. The measurements were manually reviewed and corrected if necessary. In temporospatial characterization with real-time cardiac MRI, the end-diastole and end-systole images were manually selected and segmented for ROI definition (Fig. 6). Temporal periodicity and spatial coherence were measured with the Fourier transform and correlation analysis tools in MATLAB, as detailed in the following subsection.

### Temporospatial characterization of LV and RV wall motion

Each set of real-time images consisted of cardiac images acquired from the same anatomy on the short-axis plane of the heart and at different times over a series of sequential cardiac cycles. At each spatial location, the image data along the time dimension provided a time-series signal for visualizing dynamic changes induced by local cardiac motion. As illustrated in Fig. 6, temporospatial characterization sought to analyze time-series signals temporally and spatially with Fourier transform^18^ and correlation analysis^19^ for measuring two temporospatial indices, temporal periodicity and spatial coherence. To that end, image segmentation was performed to delineate the endocardial boundaries in the end-diastole and end-systole images. By subtracting the end-systole segmentation from the end-diastole segmentation, two ROIs were defined respectively in the LV and RV. Within each ROI, the time-series signals were spatially averaged to generate a reference wall-motion signal that was in turn fed into a high-pass filter to suppress low-frequency components. Here the reference wall-motion signal would vary along the time with both cardiac and respiratory motion. Because respiration was slower than heartbeats, the low-frequency signal components should be associated with respiratory motion and the high-frequency components with cardiac motion. By suppressing the low-frequency components, the high-pass filtering prevented respiratory motion from dominating the reference wall-motion signal. This allowed the use of Fourier transform^18^ and correlation analysis^19^ to measure temporal periodicity and spatial coherence without considerable interference from respiration, as detailed below.

With the application of a Fourier transform^18^, the reference wall-motion signal was transformed into frequency domain. As ROI gave an estimate of the region within which ventricular walls moved primarily during systolic contraction and diastolic relaxation, the Fourier transform generated a frequency spectrum of globally averaged ventricular wall motion within the ROI. Due to pseudo-periodicity of heartbeats, this frequency spectrum typically had a strong spectral peak around the frequency corresponding to the average heart rate. This peak frequency was defined as the cardiac frequency. Then the temporal periodicity of ventricular wall motion was calculated by:

1 $$ Temporal\, Periodicity = \frac{{\sqrt {\left| {S\left( {f_{c} } \right)} \right|^{2} + \left| {S( - f_{c} )} \right|^{2} } }}{{\sqrt {\mathop \sum \nolimits_{n} \left[ {\left| {S(f_{n} )} \right|^{2} + \left| {S( - f_{n} )} \right|^{2} } \right]} }}, $$

where *S*(*f*_*n*_) represents the Fourier transform at an arbitrary frequency *f*_*n*_ with *n* = 0, 1, 2, …, and *S*(*f*_*c*_) represents the Fourier transform at the cardiac frequency *f*_*c*_. In this equation, the numerator gives the root sum square of the cardiac frequency components and the denominator is the root sum square of all the frequency components. It should be noted that, as real-time images are complex, the frequency spectrum of the reference wall-motion signal is not conjugate symmetric and the calculation must take into account the spectral components at both positive and negative frequencies. Equation (1) indicates that temporal periodicity is higher if the cardiac frequency components are more dominant in the Fourier transform.

A Pearson correlation coefficient^19^ was used to measure the linear correlation between the reference wall-motion signal and every time-series signal at different spatial locations, providing a correlation map over the entire anatomy. The spatial coherence of LV or RV wall motion was calculated from the correlation map as follows:

2 $$ Spatial\, Coherence = \frac{1}{M}\mathop \sum \limits_{{\left( {x, y} \right) \in VA}} R\left( {x,y} \right), $$

where *R*(*x*, *y*) is the Pearson correlation coefficient between the LV or RV reference wall-motion signal and the time-series signal at a spatial location (*x*, *y*), *VA* represents the ventricular anatomy defined by LV and RV segmentation at the end of diastole, and *M* gives the total number of voxels within the ventricular anatomy. Equation (2) implies that spatial coherence is higher if more time-series signals within the ventricular anatomy are temporally correlated to the reference wall-motion signal, i.e., if LV or RV wall motion spreads more widely over the ventricular anatomy.

### Statistical analysis

Volumetric and temporospatial indices were investigated statistically with a one-tailed *t*-test^35^_ENREF_33_ENREF_33: First, the t-test was run to compare the exercising- and the resting-state measurements of each index in the healthy volunteers. This was to investigate whether an index would be effective for detecting the exercise-induced increase of ventricular performance. Second, the t-test was run to compare the resting-state measurements of each index in the HF patients and those in the healthy volunteers. This was to investigate whether an index would be effective for detecting the disease-related decrease of ventricular performance. In addition, we evaluated the correlation between temporospatial indices and volumetric indices with a Pearson correlation coefficient^19^.

In the statistical investigation, an Anderson–Darling test^36^ was used to assess data normality before the t-test. The statistical results were presented with box plots that provide the median, 25th and 75th percentiles of the index measurements. The data summary was shown as mean ± standard deviation. The values of *P* < 0.05 were considered to be statistically significant. All the statistics were carried out in MATLAB.

## Data Availability

The datasets generated and/or analyzed during this study are available upon request.

## Abbreviations

MRI: Magnetic resonance imaging
HF: Heart failure
ECG: Electrocardiogram
LV: Left ventricle
RV: Right ventricle
EDV: End-diastolic volume
ESV: End-systolic volume
SV: Stroke volume
EF: Ejection fraction
ROI: Region of interest
